# Muscat Flavor in Grapevine: A Digital PCR Assay to Track Allelic Variation in *VvDXS* Gene

**DOI:** 10.3390/genes12050747

**Published:** 2021-05-16

**Authors:** Caterina Morcia, Giorgio Tumino, Stefano Raimondi, Anna Schneider, Valeria Terzi

**Affiliations:** 1 Council for Agricultural Research and Economics, Research Centre for Genomics and Bioinformatics, Via San Protaso 302, 29017 Fiorenzuola d’Arda, Italy; caterina.morcia@crea.gov.it; 2 Wageningen UR Plant Breeding, Wageningen University & Research, Droevendaalsesteeg 1, 6708 PB Wageningen, The Netherlands; giorgio.tumino@wur.nl; 3 Institute for Sustainable Plant Protection, National Research Council of Italy, Strada delle Cacce 73, 10135 Torino, Italy; stefano.raimondi1978@gmail.com (S.R.); anna.schneider@ipsp.cnr.it (A.S.)

**Keywords:** aroma, digital PCR, Muscat, VvDXS

## Abstract

The aroma of grapes and derived wines has long been one of the major traits considered in the selection of grapevine varieties through the centuries. In particular, Muscat aromatic grapes have been highly appreciated and widespread since ancient times. Monoterpenes are the key compounds responsible for the Muscat flavor. A major QTL affecting monoterpene level has been found to co-localize with the 1-deoxy-D-xylulose 5-phosphate synthase (*VvDXS*) gene, encoding for the 1-deoxy-D-xylulose 5-phosphate synthase enzyme involved in the plastidial pathway of terpene biosynthesis. In more detail, a single nucleotide polymorphism (SNP 1822) in the coding region of the gene causes a “gain of function” mutation, which is involved in Muscat flavor. In this work, we have developed a digital PCR-based assay to target allelic variations in the *VvDXS* gene, SNP1822, with the aim to propose a fast and sensitive analytical tool for targeting Muscat-flavored grapevine genotypes. The assay accurately predicts the genetic structure at 1822 SNP, critical for the development of the aroma in the great majority of Muscats. In the case of grapes in which the aromatic component is due to mutations other than SNP 1822 (e.g., Chasselas Musqué and Chardonnay Muscat), further specific assays can be developed.

## 1. Introduction

### 1.1. Muscat Flavor

The aroma of grapes and derived wines, intended as the olfactory component of flavor, has long been of great importance and interest in the world of viticulture and oenology and one of the major traits considered in the selection of grapevine varieties through the centuries. Moreover, the presence of aroma is greatly appreciated even for fresh grape consumption.

A wide variety of compounds contribute to the aromatic profile of grapes, the main classes include mono- and sesquiterpenes, methoxypyrazines, furan derivatives, and products of the lipoxygenase and phenylpropanoid pathways [1]. Monoterpenes are the key compounds responsible for the ‘Muscat’ flavor, and contribute to aroma only in the free form, being inactive when bound to glucosidic moieties. They are present in the first developmental stage of berries in most varieties, but their level dramatically declines below detectable levels during veraison. This is not true for Muscats, in which high concentrations of linalool, geraniol, nerol, alfa-terpineol, hotrienol, and other free monoterpenes are conserved in ripe berries. Muscat varieties, while presenting different ampelographic characteristics, have such a unique scent in common. From a historical point of view, such aromatic grapes have been highly appreciated and verisimilarly widespread throughout the Mediterranean basin since ancient times [2,3,4]. Starting from the Middle Ages, the grapes and their derived aromatic wines were called Moscatella, Moscatello, or Moscato, whose etymology is traced back to the late Latin term “muscus”, or musk—essence extracted from some mammals and used as a base for precious perfumes.

### 1.2. VvDXS Gene

Several QTL studies have been carried out to elucidate the genetic bases of Muscat flavor, highlighting how the trait is verisimilarly controlled by a reduced number of loci with strong effects [5,6]. A crucial step in understanding the genetics behind Muscat flavor has been the identification of a major QTL affecting monoterpene levels carried out by Battilana et al. [7]. Such a QTL co-localizes with the 1-deoxy-D-xylulose 5-phosphate synthase (*VvDXS*) gene, encoding for the DXS enzyme involved in the plastidial pathway of terpene biosynthesis [8]. In more detail, DXS is the first enzyme involved in the non-mevalonate pathway of isopentenyl-5-pyrophosphate (IPP) biosynthesis. A particular *VvDXS* allele is responsible for the increasing level of IPP, which is the precursor of linalool, nerol, and geraniol. An excess of isoprenoids, in turn, determines a high level of monoterpenes. In a subsequent association study [9], the allelic variation in the *VvDXS* gene was evaluated in aromatic and non-aromatic varieties, finding a single nucleotide polymorphism (SNP 1822) in the coding region of the gene, causing a “gain of function” mutation. Due to such substitution in the coding sequence, the lysin at position 284 is replaced by an asparagine in nearly all of the Muscat-flavored varieties. Analytical protocols have been proposed to target allelic variation in SNP1822, including high resolution melting, minisequencing, and a cleaved amplified polymorphic sequence system [10,11]. No digital PCR-based assay has been developed until now for this target.

### 1.3. Digital PCR

Digital PCR (dPCR) is an innovative technique aimed at identifying and quantifying a target sequence with very high levels of specificity, sensitivity, and precision. The peculiarity of this technology lies in the fact that the analytical sample is divided into numerous compartments in which independent PCR reactions take place. The compartmentalization can be realized with different technical solutions, ranging from the use of chip hosting micro-wells to the generation of emulsions. The average number of molecules per partition is estimated using Poisson statistics, and then converted into concentration by dividing by partition volume. Digital PCR has increasing applications in plant science, as recently reviewed by Morcia et al. [12]. This technology, widely used in medical research and diagnostics, is currently growing in plant science. The majority of the applications reported have been developed for the identification and quantification of genetically modified plants in food and feed. Moreover, dPCR is a useful tool for the characterization of transgenic lines obtained with classical or innovative technologies of genetic modification. The applications of dPCR are relevant in the sectors of plant and soil microbiology for the diagnosis of plant pathogens and for the quantification of specific soil microorganisms. Plant species quantification in agri-food and feed production chains can be efficiently performed with dPCR, and very recently, an example of the application of this technology to traceability at the variety level has been proposed in the pasta production chain [13]. Digital PCR can be applied to target specific mutations through allelic variation and low-frequency and/or rare SNP detection [14].

In this work, we have developed a dPCR-based assay to target allelic variations in the *VvDXS* gene, SNP1822, demonstrated to be related to the Muscat flavor development. The final aim was to propose a fast and sensitive analytical tool to target Muscat-flavored grapevine genotypes.

## 2. Materials and Methods

### 2.1. Grapevine Varieties

The following varieties were considered in this study: Aleatico, Chardonnay non-Muscat clone, Chardonnay Muscat clone, Chasselas Musqué (Muscat variant of Chasselas), Early Muscat, Malvasia nera, Moscato bianco (Muscat à petits grains blancs), Moscato di Scanzo, Muscat reine des vignes, Muscat Susanna, and Muskat Vostochnyi. All of the above listed varieties exhibit a Muscat flavor, except for the non-Muscat clone of Chardonnay. This set of genotypes were selected as representatives of the different variants at the SNP 1822, *VvDXS* gene, as inferred from previous sequencing characterizations [10].

### 2.2. DNA Extraction

Young leaf tissues were sampled, and their genomic DNA was extracted using a CTAB-based buffer followed by chloroform extraction. The evaluation of the quality and quantity of the extracted DNA was performed using a Qubit™ fluorometer in combination with the Qubit™ dsDNA BR Assay kit (Invitrogen by Thermo Fisher Scientific, Monza, Italy).

### 2.3. Amplification and Sequencing

The DNAs were amplified with the following primers:
DXS8fwCAACAACGTCATTGCTGTCATAG (5′-3′)DXS8rwGCTAGACAGAACAGGTAAGATTTC (5′-3′)

The amplification products were purified and sequenced according to Emanuelli et al. [9].

### 2.4. Chip Digital PCR

Primers and minor groove binding-TaqMan (MGB) probes were designed on the ***VvDXS1*** gene sequence, focusing on SNP 1822. The choice of MGB probes was based on the highly stable interaction between the MGB probe and the target, which increases the T_m_ of the probe, avoiding the amplification of non-specific products and providing a more accurate allelic discrimination [15]. The two alleles, T/G, at 1822 SNP are discriminated by different fluorophores; hydrolysis probes specific to the wild type (G) and mutated (T) alleles are conjugated to VIC and FAM, respectively. The forward and reverse primers are identical. The assay has been designed using the Custom TaqMan SNP Genotyping Assay, not human, procedure (Thermo Fisher Scientific, Monza, Italy). In more detail, Primer Express Software 3.0, TaqMan MGB Allelic Discrimination option was used to design probes and primers by treating the single-nucleotide substitution as an SNP in the software. The system generates, if possible, two allele-specific MGB probes conjugated with fluorescent dyes, FAM and VIC, and the forward and reverse primers. Typically, the amplicon size is <200 bp, and the primers and probes are available as Assay ID ANU7GME, Catalog n. 4332077 (Thermo Fisher Scientific, Monza, Italy).

Chip digital PCR was performed using QuantStudioTM 3D Digital PCR System (Applied Biosystems by Life Technologies, Monza. Italy). The reaction mixture was prepared in a final volume of 16 µL, consisting of 8 µL QuantStudioTM 3D Digital PCR 2X Master Mix, 0.72 µL of each primer at 20 µM (final concentration 900 nmol), 0.32 µL of FAM and VIC-MGB probes at 10 µM (final concentration 200 nmol), 2 µl of DNA (40 ng/µL), and nuclease free-water. Additionally, a negative control with nuclease-free water as a template was added. A total volume of 15 µL reaction mixture was loaded onto the QuantStudioTM 3D Digital PCR chips using QuantStudioTM 3D Digital chip loader, according to the manufacturer’s protocol. Amplifications were performed in a ProFlexTM 2Xflat PCR System Thermocycler (Applied Biosystems by Life Technologies, Monza, Italy) under the following conditions:

- 96 °C for 10 min;
- 55 °C for 2 min and 98 °C denaturation for 30 s, repeated for 45 cycles;
- 60 °C for 2 min.

End-point fluorescence data were collected in QuantStudioTM 3D Digital PCR Instrument and the files generated were analyzed using the cloud-based platform QuantStudioTM 3D AnalysisSuite dPCR software, version 3.1.6.

## 3. Results and Discussion

A new dPCR assay aimed at tracking Muscat flavored grapevine genotypes has been developed and validated on the set of aromatic and non-aromatic grapevine varieties listed in the Materials section. The assay was designed to discriminate T/G polymorphism at SNP 1822: such a point mutation, occurring in the *VvDXS* gene, has been associated with Muscat flavor development [9].

**Figure 1:**
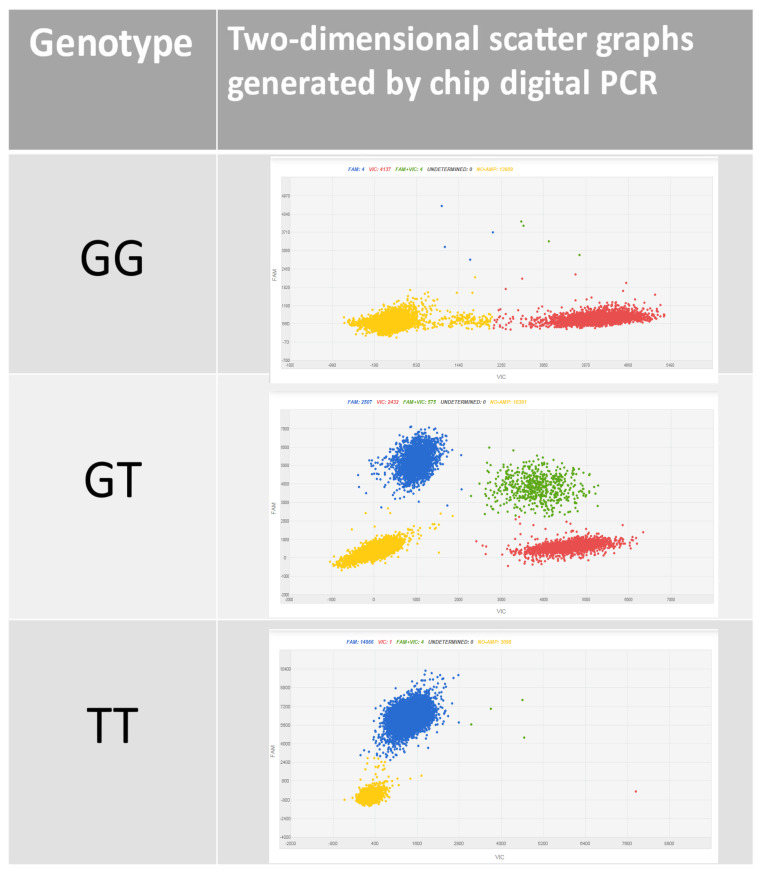
Two-dimensional scatter graphs generated by the chip digital PCR (cdPCR) analysis of Muscat-flavored and non-Muscat-flavored grapevine samples. The samples were compartmentalized, and each partition can fall into one of three possible classes: negative partitions, which contain no DNA molecules (yellow dots); positive partitions, containing a single DNA molecule (blue or red dots); and partitions hosting more than one single DNA molecule, containing positive signals for both targets (green dots). Accordingly, G/G homozygous genotypes show VIC marked amplifications (red cloud) and T/T homozygous genotypes show FAM marked amplifications (blue cloud), whereas G/T heterozygous genotypes showed both FAM and VIC signals (blue and red clouds plus the green one, due to the co-amplifications of T and G). All of the patterns have a yellow cloud, due to DNA-empty wells.

The dPCR assay, when applied to the analysis of Muscat and non-Muscat grapevine varieties, produced three classes of amplification patterns, depending on the allelic arrangement, as schematically reported in Figure 1.

The dPCR assay designed was used to categorize the grapevine varieties representatives of the different variants at SNP 1822, and the results obtained are reported in Table 1, in comparison with the sequencing data at the same locus.

The two Chardonnay clones and Chasselas Musqué showed VIC signals only, with a 0% percentage of FAM signals, suggesting their G/G homozygosity, as confirmed by the sequencing. On the contrary, Malvasia nera and Muskat Vostochnyi had 100% FAM signals due to their T/T homozygous genetic makeup. All the other varieties were heterozygous G/T and showed a mean FAM percentage of 50.6 ± 1.5. Such a percentage is congruent with the sequence data.

It should be noted that both Chasselas Musqué and Chardonnay Muscat are classified by the dPCR assay as homozygous GG to SNP 1822, despite being two aromatic clones. This result is congruent with the findings of Emanuelli et al. [9,10], which explain how the aroma of these two clones is due to mutations present in positions other than SNP 1822 in the gene coding for the VvDXS1 protein. In detail, Chasselas Musqué has a mutation at SNP 1917, while Chardonnay Muscat is mutated at SNP 1784 of the *VvDXS1* gene sequence. These mutations give rise to non-neutral amino acid changes in the VvDXS1 protein, resulting in the variation of the monoterpene content and aroma development. From the data obtained, it can be concluded that the assay accurately predicts the genetic structure at 1822 SNP, critical for the development of the aroma in Muscat grapes. The assay can therefore be used as a predictor of the Muscat flavor of a grape variety. Its sensitivity is discriminate between homozygosity and heterozygosity at the 1822 SNP, VvDXS locus, and can be a tool in breeding programs for new aromatic genotypes. In the present study, a chip-based instrument was used, characterized by low costs and moderate output in terms of the number of analytical samples that can be processed in a unit of time. However, other platforms are currently available, and can be divided into different types according to the compartmentalization methods, which can be active (a mechanical aid helps the compartment formation), passive (based on fluidic effects), or droplet-based (aqueous droplets act as microreactors). Different platforms have been evaluated in terms of effectiveness in quantifying DNA copy number and, despite some peculiarities, they have all been found comparable [16]. Moreover, interest in developing multiple target detection, improving workflow, and reducing analytical times and costs is increasing. Experimental considerations to ensure the accurate quantification of multiple targets have been reviewed by Whale et al. [17], and practical applications for GMO detection have already been developed [18,19].

In the recent past, other analytical approaches have been proposed for identifying nucleotide polymorphisms functionally related to Muscat aroma, namely high-resolution melting, minisequencing, and cleaved amplified polymorphic sequence systems [10]. The main advantages of dPCR are in its reduced costs, in the robustness of the data obtained, and in the independence from reference samples. Even the greater resilience of this technique is remarkable compared to the other PCR-based technologies and to inhibitors that are commonly found in the samples of seeds, plant materials, soils, and wastewater [20]. All of these technical features make digital PCR-based approaches feasible for grapevine precision breeding [21,22], targeting favorable haplotypes for an important quality-related trait, such as aroma.

## Figures and Tables

**Table 1:** Varietal names, Muscat flavor (according to [10]), the 1822 SNP sequences at the *VvDXS* gene, and dPCR data obtained expressed as a percentage of FAM copies/µL on the total (FAM + VIC) copies/µL.

Variety	Muscat Flavor	Sequence at 1822 SNP	FAM Percentage
Chardonnay Muscat clone	+	G/G	0
Chardonnay non-Muscat clone	−	G/G	0
Aleatico	+	G/T	49.8
Chasselas Musqué	+	G/G	0
Early Muscat	+	G/T	50
Moscato bianco	+	G/T	52.9
Moscato di Scanzo	+	G/T	49.6
Muscat reine des vignes	+	G/T	50
Muscat Susanna	+	G/T	49.3
Malvasia nera	+	T/T	99.9
Muskat Vostochnyi	+	T/T	100

## Data Availability

Data is contained within the article.
